# Association of Race and Ethnicity With Triage Emergency Severity Index Scores and Total Visit Work Relative Value Units for Emergency Department Patients

**DOI:** 10.1001/jamanetworkopen.2022.31769

**Published:** 2022-09-14

**Authors:** Joshua W. Joseph, Alden M. Landry, Maura Kennedy, Da’Marcus Eugene Baymon, Alice K. Bukhman, Noémie Elhadad, León D. Sanchez

**Affiliations:** 1 Department of Emergency Medicine, Brigham and Women’s Hospital, Boston, Massachusetts; 2 Department of Emergency Medicine, Harvard Medical School, Boston, Massachusetts; 3 Department of Emergency Medicine, Beth Israel Deaconess Medical Center, Boston, Massachusetts; 4 Department of Emergency Medicine, Massachusetts General Hospital, Boston; 5 Department of Biomedical Informatics, Columbia University, New York, New York; 6 Department of Computer Science, Columbia University, New York, New York

## Abstract

This cross-sectional study evaluates the association of race and ethnicity with triage Emergency Severity Index scores and total work relative value units for emergency department (ED) patients.

## Introduction

Previous literature on emergency department (ED) patients has suggested that nurses assign lower-acuity triage scores to non-White patients and that physicians order fewer diagnostic interventions for them.^1,2^ A potential cause of this phenomenon is a higher rate of nonurgent symptoms, owing to disparities in socioeconomic status and access to primary care.^3^ It is less clear whether these discrepancies persist for specific, urgent chief symptoms.

## Methods

In this retrospective cross-sectional study examining adult patients at an academic, urban ED in the Northeastern United States with an average of 55 000 visits annually, we investigated the triage Emergency Severity Index (ESI) scores (range, level 1 [most urgent] to level 5 [least urgent]) and work relative value units (wRVUs) associated with patients’ visits for the 5 most common acute chief symptoms (abdominal pain, chest pain, dyspnea, nausea or vomiting, and falls). Owing to the association of diagnostic testing and procedures with wRVUs, they served as a proxy for the intensity of a patient’s diagnostic workup. All unique patient visits between July 1, 2016, and March 1, 2020, were included in the study. Data collected at triage included self-reported race and ethnicity (American Indian or Alaska Native, Asian, Black, Hispanic, other race or ethnicity [the registration process at the institution allows patients to self-identify race as “other” in cases where they do not feel that the provided categories adequately describe their racial and ethnic identity], and White). The study was granted an exemption from review and from the use of informed consent by the Beth Israel Deaconess Medical Center institutional review board owing to the use of deidentified, aggregated data. This study followed the Strengthening the Reporting of Observational Studies in Epidemiology (STROBE) reporting guideline.^4^

Differences in ESI scores and wRVUs between self-identified racial and ethnic groups were evaluated using stratified independent *t* tests, with mean values and 95% CIs as descriptive statistics. An overall significance level of *P* < .001 was used to correct for 5 sets of comparisons across racial and ethnic groups for 5 chief symptoms. To capture differences in physician evaluation independent of testing typically ordered from nursing triage for patients with acute ESI scores (levels 1 and 2), we conducted a secondary analysis for patients assigned ESI level 3 only. Analysis was performed in Python, version 3.9 (Python Software Foundation) using the SciPy library.^5^

## Results

A total of 204 064 patients (109 519 women [53.7%]; median age, 55 years [IQR, 25-69 years]) were included in the study. Baseline patient characteristics are described in Table 1. A total of 0.6% patients were American Indian or Alaska Native, 4.6% were Asian, 22.4% were Black, 8.4% were Hispanic, 57.3% were White, and 6.7% were other race or ethnicity. The 5 most common chief symptoms (abdominal pain, 13.5%; chest pain, 8.2%; dyspnea, 7.2%; nausea or vomiting, 6.5%; and falls, 6.4%) represented urgent reasons for ED visits, generally associated with ESI scores of 2 and 3, representing high-risk conditions needing expedited evaluation or stable patients needing multiple resources.

**Table 1.** Characteristics of the Study Population

Characteristic	Patients, No. (%)								
	All (N = 204 064)	Male (n = 94 545 [46.3%])	Female (n = 109 519 [53.7%])	American Indian or Alaska Native (n = 1299 [0.6%])	Asian (n = 9398 [4.6%])	Black (n = 45 748 [22.4%])	Hispanic (n = 17 134 [8.4%])	Other (n = 13 635 [6.7%])	White (n = 116 850 [57.3%])
Age, median, (IQR), y	55 (25-69)	55 (37-68)	54 (33-70)	45 (28-64)	47 (26-67)	52 (35-65)	49 (34-63)	45 (29-64)	58 (38-72)
Female	109 519 (53.7)	0	109 519 (100)	672 (51.7)	5256 (55.9)	27 037 (59.1)	9788 (57.1)	7122 (52.2)	59 689 (51.1)
ESI score, median, (IQR)	3 (2-3)	3 (2-3)	3 (2-3)	3 (2-3)	3 (2-3)	3 (2-3)	3 (2-3)	3 (2-3)	3 (2-3)
Abdominal pain	27 557 (13.5)	10 380 (11.0)	17 177 (15.7)	189 (14.5)	1280 (13.6)	5836 (12.8)	2875 (16.8)	1696 (12.4)	15 681 (13.4)
Chest pain	16 747 (8.2)	7983 (8.4)	8764 (8.0)	104 (8.0)	641 (6.8)	4509 (9.9)	1730 (10.1)	956 (7.0)	8807 (7.5)
Dyspnea	14 742 (7.2)	6681 (7.1)	8061 (7.4)	75 (5.8)	570 (6.1)	3421 (7.5)	1111 (6.5)	800 (5.9)	8765 (7.5)
Nausea or vomiting	13 275 (6.5)	4543 (4.8)	8732 (8.0)	92 (7.1)	609 (6.5)	3126 (6.8)	1293 (7.5)	800 (5.9)	7355 (6.3)
Falls	12 976 (6.4)	5442 (5.8)	7534 (6.9)	47 (3.6)	442 (4.7)	1726 (3.8)	697 (4.1)	716 (5.3)	9348 (8.0)

Abbreviation: ESI, Emergency Severity Index.

For all 5 of the chief symptoms that we evaluated in this study, White patients were assigned significantly more acute mean ESI scores from triage than Black and Hispanic patients (eg, abdominal pain: White patients, 2.71 [95% CI, 2.70-2.72]; Black patients, 2.82 [95% CI, 2.80-2.83]; and Hispanic patients, 2.80 [95% CI, 2.79-2.82]) (Table 2). White patients also had a greater mean number of wRVUs associated with their visit relative to Black and Hispanic patients for all 5 of the chief symptoms we evaluated in this study (eg, chest pain: White patients, 3.56 [95% CI, 3.54-3.58]; Black patients, 3.35 [95% CI, 3.32-3.38]; and Hispanic patients, 3.35 [95% CI, 3.32-3.38]). This difference remained when controlling for lower acuity scores assigned from triage. Similar differences were seen across racial and ethnic categories for the wRVUs associated with visits for abdominal pain and in ESI scores for patients self-identifying as Asian or other race or ethnicity with chest or abdominal pain. No differences were seen in insurance status, mode of arrival, or primary language.

**Table 2.** Emergency Severity Index and Visit Work Relative Value Units Differences Between Racial and Ethnic Groups

Chief symptom	American Indian or Alaska Native patients	Asian patients	Black patients	Hispanic patients	Patients of other race or ethnicity	White patients
Work relative value units						
Abdominal pain	3.33 (3.16-3.50)^a^	3.37 (3.30-3.43)^a^	3.34 (3.31-3.37)^a^	3.34 (3.30-3.39)^a^	3.44 (3.38-3.50)^a^	3.75 (3.73-3.77)
Chest pain	3.25 (3.06-3.45)	3.39 (3.30-3.47)	3.35 (3.32-3.38)^a^	3.32 (3.27-3.36)^a^	3.43 (3.35-3.50)	3.56 (3.54-3.58)
Dyspnea	3.94 (3.59-4.29)	3.99 (3.87-4.12)	3.84 (3.79-3.88)^a^	3.80 (3.72-3.88)^a^	4.14 (4.02-4.25)	4.09 (4.07-4.12)
Nausea or vomiting	3.53 (3.29-3.78)	3.42 (3.32-3.52)^a^	3.53 (3.49-3.58)^a^	3.38 (3.32-3.44)^a^	3.39 (3.31-3.47)^a^	3.73 (3.70-3.75)
Falls	3.71 (3.19-4.24)	3.54 (3.37-3.71)	3.16 (3.09-3.23)^a^	3.41 (3.27-3.54)^a^	3.83 (3.80-3.87)	3.83 (3.80-3.87)
Emergency Severity Index score, mean (95% CI)						
Abdominal pain	2.78 (2.72-2.84)	2.81 (2.78-2.83)^a^	2.82 (2.80-2.83)^a^	2.80 (2.79-2.82)^a^	2.75 (2.73-2.77)	2.71 (2.70-2.72)
Chest pain	2.49 (2.39-2.59)	2.44 (2.40-2.48)^a^	2.49 (2.47-2.50)^a^	2.48 (2.45-2.50)^a^	2.41 (2.37-2.45)^a^	2.33 (2.32-2.35)
Dyspnea	2.31 (2.17-2.44)	2.22 (2.17-2.27)	2.28 (2.26-2.30)^a^	2.26 (2.23-2.30)^a^	2.11 (2.06-2.16)	2.14 (2.13-2.16)
Nausea or vomiting	2.74 (2.63-2.85)	2.74 (2.70-2.77)	2.77 (2.75-2.79)^a^	2.78 (2.76-2.80)^a^	2.75 (2.72-2.78)	2.69 (2.68-2.70)
Falls	2.62 (2.40-2.84)	2.70 (2.64-2.77)	2.75 (2.72-2.78)^a^	2.68 (2.63-2.74)^a^	2.18 (2.11-2.25)	2.47 (2.46-2.48)

^a^ Significant differences relative to White patients.

## Discussion

Our study results suggest that significant racial and ethnic disparities exist in the ESI scores assigned to patients during nursing triage evaluation and in the intensity of services provided during physician evaluation for patients presenting with the same acute chief symptoms. When nurses assign White patients more acute ESI scores at triage, they may also order diagnostic tests prior to a physician’s involvement, leading to downstream increases in wRVUs. Additional decisions made at triage associated with race, ethnicity, and socioeconomic status, such as assignment to a room or hallway bed, may also influence physicians.^6^ However, our findings suggest that when controlling for triage, significant racial and ethnic disparities persist in physicians’ evaluations, particularly for Black and Hispanic patients.

This study has several significant limitations, including a smaller proportion of patients identifying as Asian and American Indian or Alaska Native and a heterogenous other race or ethnicity category, limiting our ability to draw conclusions about these populations. More work is needed to determine where in the triage and physician evaluation processes these disparities arise and what can be done to remedy them.
